# Outcomes of phacoemulsification combined with ab interno trabeculotomy: stratified by incision extent, glaucoma severity, and baseline number of medications

**DOI:** 10.1038/s41433-026-04749-x

**Published:** 2026-07-22

**Authors:** Takashi Nishida, Takuhei Shoji, Masaki Tanito

**Affiliations:** 1 Koedo Eye Institute, Kawagoe, Saitama Japan; 2 Hamilton Glaucoma Center, Shiley Eye Institute, Viterbi Family Department of Ophthalmology, University of California San Diego, La Jolla, CA USA; 3 Department of Ophthalmology, Saitama Medical University, Saitama, Japan; 4 Department of Ophthalmology, Shimane University Faculty of Medicine, Izumo, Shimane Japan

**Keywords:** Glaucoma, Outcomes research

## Abstract

**Objectives:**

To evaluate outcomes of phacoemulsification plus Tanito microhook (TMH) trabeculotomy and assess differences by incision extent, glaucoma severity, and baseline medications classes.

**Methods:**

Retrospective cohort of 880 open-angle glaucoma eyes (119 exfoliation glaucoma, 761 primary open-angle glaucoma) undergoing phaco-TMH. Success was defined using the American Academy of Ophthalmology (AAO) minimally invasive glaucoma surgery (MIGS) endpoint for cataract-combined procedures ( ≥ 1 medication reduction without intraocular pressure [IOP] increase, or ≥20% IOP reduction to ≤21 mmHg) with no additional glaucoma surgery, hypotony, or loss of light perception. Prespecified alternative IOP-defined success definitions were also evaluated. Outcomes were stratified by incision extent ( < 6 and ≥6 clock hours), baseline glaucoma severity (early, moderate, advanced), and baseline medications classes (0–1, 2, ≥3). Time-to-failure was evaluated using Kaplan-Meier analysis and multivariable Cox proportional hazards models adjusted for surgical year and baseline covariates.

**Results:**

Mean (SD) IOP decreased from 17.2 (4.9) mmHg at baseline to 13.1 (3.5) at 12 months and 13.1 (3.9) at 24 months, and medications decreased from 2.6 (1.3) to 2.0 (1.3) at 12 and 24 months. Two-year cumulative success was 59.0% (95% CI, 54.4 to 63.2) by AAO MIGS criteria. In the multivariable Cox model, ≥6 clock hours was not significantly associated with surgical failure compared with <6 clock hours (adjusted HR, 1.14; 95% CI, 0.85 to 1.53; *P* = 0.367). Higher baseline medication burden was associated with worse success across IOP-defined criteria.

**Conclusions:**

In patients undergoing phacoemulsification combined with TMH trabeculotomy, IOP and medication burden improved through 2 years. Surgical success did not differ by incision extent or baseline glaucoma severity, whereas higher baseline medication burden was associated with worse IOP-defined success.

## Introduction

Minimally invasive glaucoma surgery (MIGS) procedures combined with cataract extraction are commonly performed to reduce intraocular pressure (IOP) and medication burden with an acceptable safety profile [1–4]. Standardised endpoint reporting has been recommended to improve interpretability across studies, including medication reduction without IOP worsening and IOP-based thresholds without escalation of therapy [5].

Angle-based, incisional MIGS aim to enhance conventional aqueous outflow by incising or excising the trabecular meshwork and the inner wall of Schlemm’s canal under gonioscopic visualisation. Across commonly used techniques, the treated arc varies by device and surgical approach and is often selected at the surgeon’s discretion. For example, excisional goniotomy with the Kahook Dual Blade typically removes approximately 3–5 clock hours of trabecular meshwork [6], whereas ab interno trabeculotomy with Trabectome commonly treats approximately 60°–120° of angle [7]. In contrast, catheter-based approaches can intentionally extend treatment more broadly, using the OMNI surgical system, gonioscopy-assisted transluminal trabeculotomy, or suture trabeculotomy [8, 9]. Because incision extent may reflect surgeon preference and intraoperative considerations as much as a planned efficacy strategy, real-world comparative effectiveness may be more influenced by baseline disease characteristics, including preoperative medication burden and severity, than by the intended arc alone.

Tanito microhook (TMH) trabeculotomy is an ab interno trabeculotomy technique performed under gonioscopic visualisation [10–12]. In this study, we evaluated outcomes of phacoemulsification combined with TMH trabeculotomy and compared outcomes by incision extent, baseline glaucoma severity, and baseline number of glaucoma medications.

## Methods

### Study design and setting

This retrospective cohort study included consecutive eyes undergoing phacoemulsification with intraocular lens implantation combined with TMH trabeculotomy (phaco-TMH). Eligible eyes were required to have at least 3 months of postoperative follow-up. The cohort included 880 eyes (119 exfoliation glaucoma and 761 primary open-angle glaucoma). At Matsue Red Cross Hospital (surgeon MT), the original Tanito microhook (TMH) was used, whereas at Koedo Eye Institute (surgeon TS), the Shoji edition of TMH was used. Because our prior propensity score-matched analysis using baseline age, intraocular pressure (IOP), number of glaucoma medications, and visual field mean (VF) deviation (MD) showed similar results between two TMH devices, we analysed the full eligible cohort without matching in the present study. The study was approved by the Institutional Review Board of Shimane University (KS20200517-1). The study adhered to the tenets of the Declaration of Helsinki. Written informed consent was obtained from all participants for undergoing surgery. With regard to participation in the study, the study information was disclosed at each participating institution, and patients were given the opportunity to opt out in lieu of providing written consent.

### Surgical procedure

TMH trabeculotomy was performed either before or after phacoemulsification under gonioscopic visualisation depending on the surgeon’s preference. A cohesive ophthalmic viscosurgical device was used in all cases; Provisc (sodium hyaluronate 1%; Alcon Japan, Tokyo, Japan) and/or Opegan Hi (sodium hyaluronate 1%; Santen Pharmaceutical, Osaka, Japan) was chosen at the surgeon’s discretion. A microhook (straight, right-angled, or left-angled; Inami & Co., Ltd, Tokyo, Japan) was introduced through the main incision or a side port, and the trabecular meshwork and Schlemm canal were incised for at least 3 clock hours. In eyes undergoing wider trabeculotomy, a dual-sided approach was used to access a broader portion of the angle, allowing treatment of up to approximately 8–9 clock hours. When trabeculotomy was performed through a temporal approach alone, treatment was generally limited to the nasal angle, resulting in a smaller incision extent. Incision extent was recorded from the intraoperative surgical record as the surgeon’s estimated treated clock hours under gonioscopic visualisation. At the conclusion of surgery, the viscoelastic was removed, corneal incisions were hydrated, and subconjunctival dexamethasone was administered. Postoperatively, patients received topical antibiotics and corticosteroids per standard protocol.

### Exposures and stratification variables

1. Incision extent was categorised into two groups ( < 6 clock hours and ≥6 clock hours). Incision extent was largely determined by the calendar period and surgeon practice pattern. Surgeon M.T. typically performed ≥6 clock hours until July 2017 and <6 clock hours thereafter, whereas surgeon T.S. most often selected ≥6 clock hours through December 2022 and <6 clock hours from January 2023 onward.
2. Glaucoma severity was categorised by baseline 24-2 VF MD as early (>−6 dB), moderate (>−12 dB and ≤−6 dB), and advanced (≤−12 dB).
3. The number of glaucoma medications was calculated as the total count of distinct medication classes. For example, a fixed-combination latanoprost/timolol preparation was counted as two medications. For analyses, baseline number of glaucoma medication classes was categorised into three groups: 0–1, 2, and ≥3.

### Outcome

Supplementary Table 1 summarised the definitions of surgical failure criteria. Primary outcome was surgical success defined by American Academy of Ophthalmology (AAO)’s minimally invasive glaucoma surgery (MIGS) criteria for procedures combined with cataract surgery: (1) reduction of at least one glaucoma medication from baseline without an increase in IOP, or (2) IOP ≤ 21 mmHg with ≥20% reduction from baseline, without additional glaucoma surgery, laser, loss of light perception, or hypotony. Because the medication-reduction component can complicate comparisons across strata defined by baseline number of medications, subgroup comparisons of surgical success were based on IOP-defined success only. Medication reduction was therefore evaluated as a secondary outcome using longitudinal analyses of postoperative medication counts. Four additional IOP-defined failure definitions were examined as sensitivity endpoints. For criterion A, failure was defined as IOP > 18 mmHg or <20% IOP reduction from baseline. For criterion B, failure was defined as IOP > 15 mmHg or <20% IOP reduction. For criterion C, failure was defined as IOP > 18 mmHg or postoperative IOP above baseline. For criterion D, failure was defined as IOP > 15 mmHg or postoperative IOP above baseline. For all four definitions, additional glaucoma surgery or loss of light perception was also counted as failure. Antiglaucoma medication use was allowed in these qualified success definitions [13].

Postoperative complications and safety outcomes were collected from the clinical records. These included hyphaema or retained blood, hyphaema requiring treatment, IOP spike, hypotony, fibrin reaction, macular oedema, ciliary body detachment, vitreous haemorrhage, additional glaucoma surgery, loss of light perception, and best-corrected visual acuity (BCVA) loss of ≥2 lines at 12 months. Hyphaema requiring treatment included anterior chamber washout, tissue plasminogen activator use, or recorded hyphaema treatment. BCVA loss of ≥2 lines was defined as an increase in logMAR BCVA of ≥0.2 from baseline to 12 months. IOP spike was defined as postoperative IOP ≥ 30 mmHg, and hypotony was defined as postoperative IOP < 5 mmHg.

### Statistical analysis

Patient and eye characteristics were summarised as means with standard deviations for continuous variables and counts with percentages for categorical variables. Continuous variables were compared using a linear mixed model and categorical variables were compared using the chi-square test. Time-to-failure outcomes were evaluated using Kaplan-Meier survival analysis and log-rank tests. In response to potential confounding by calendar time, multivariable Cox proportional hazards models were fitted. The model included incision extent, age, sex, glaucoma diagnosis, baseline IOP, baseline 24-2 VF MD, baseline number of glaucoma medications, and surgical year. Robust standard errors were clustered by patient. Alternative exploratory IOP-defined criteria A–D were analysed as sensitivity endpoints. For the primary AAO-based endpoint, IOP- and medication-based failure was assessed from postoperative day 30 onward and required confirmation at two consecutive eligible postoperative visits. Postoperative complications were counted at the recorded time of occurrence. For the alternative IOP-based criteria A–D, IOP-based failure was assessed from postoperative day 90 onward. Eyes not meeting failure criteria were censored at the last follow-up visit. All statistical tests were two-sided, and P values less than 0.05 were considered statistically significant. No adjustments were made for multiple comparisons. Analyses were performed using Stata version 16.0 (StataCorp LLC, College Station, TX, USA).

## Results

A total of 880 eyes from 700 participants were included, including 761 eyes with primary open-angle glaucoma and 119 eyes with exfoliation glaucoma. Baseline characteristics stratified by incision extent are shown in Table 1. Overall, 402 eyes (45.7%) were classified as <6 clock hours and 478 eyes (54.3%) as ≥6 clock hours. Baseline mean (SD) age was 74.1 (8.4) years, and baseline VF MD was -10.7 (7.7) dB. Mean (SD) IOP decreased from 17.2 (4.9) mmHg at baseline to 13.1 (3.5) mmHg at 12 months and 13.1 (3.9) mmHg at 24 months. Mean (SD) number of glaucoma medications decreased from 2.6 (1.3) at baseline to 2.0 (1.3) at 12 months and 2.0 (1.3) at 24 months. The mean (SD) percent IOP reduction from baseline was 20.2 (21.2) % at 12 months and 18.7 (22.0) % at 24 months. Postoperative 12-month and 24-month IOP data were available for 458 and 232 eyes, respectively.

**Table 1:** Demographics and baseline clinical characteristics of the subjects.

Demographic characteristics	Overall	<6 clock hours	>=6 clock hours	*P* value
Participants, No.	700	283	417	
Age (years), mean (SD)	74.1 (8.4)	74.8 (8.3)	73.6 (8.5)	0.058
Sex, % female	376 (53.7%)	156 (55.1%)	220 (52.8%)	0.589
Clinical characteristics				
Eyes, No.	880	402	478	
Diagnosis, No. (%)				<0.001
POAG	761 (86.5%)	377(93.8%)	384 (80.3%)	
XFG	119 (13.5%)	25 (6.2%)	94 (19.7%)	
24-2 VF MD (dB), mean (SD)	–10.7 (7.7)	–10.4 (7.6)	–10.9 (7.8)	0.399
Disease severity by baseline 24-2 VF MD, No. (%)				0.930
Early glaucoma (MD >−6 dB)	325 (36.9%)	151 (37.6%)	174 (36.4%)	
Moderate glaucoma (−12 dB < MD ≤ −6 dB)	237 (26.9%)	108 (26.9%)	129 (27.0%)	
Advanced glaucoma (MD ≤−12 dB)	318 (36.1%)	143 (35.6%)	175 (36.6%)	
Baseline BCVA, No. (%)				0.056
20/20–20/40	609 (69.2%)	258 (64.2%)	351 (73.4%)	
20/50–20/70	159 (18.1%)	83 (20.7%)	76 (15.9%)	
20/80–20/100	39 (4.4%)	20 (5.0%)	19 (4.0%)	
20/200–20/400	49 (5.6%)	28 (7.0%)	21 (4.4%)	
<20/400	24 (2.7%)	13 (3.2%)	11 (2.3%)	
Number of medications at baseline, No. (%)				0.862
0–1 drop	228 (25.9%)	107 (26.6%)	121 (25.3%)	
2 drops	175 (19.9%)	81 (20.1%)	94 (19.7%)	
≥3 drops	477 (54.2%)	214 (53.2%)	263 (55.0%)	
Baseline IOP (mmHg), mean (SD)	17.2 (4.9)	16.5 (3.8)	17.8 (5.6)	<0.001
Baseline IOP category, No. (%)				<0.001
≤15 mmHg	333 (37.8%)	156 (38.8%)	177 (37.0%)	
>15–18 mmHg	274 (31.1%)	144 (35.8%)	130 (27.2%)	
>18–21 mmHg	154 (17.5%)	67 (16.7%)	87 (18.2%)	
>21 mmHg	119 (13.5%)	35 (8.7%)	84 (17.6%)	
Baseline number of glaucoma medications, mean (SD)	2.6 (1.3)	2.7 (1.4)	2.5 (1.2)	0.234
Site				<0.001
Matsue	347 (39.4%)	57 (14.2%)	290 (60.7%)	
Koedo	533 (60.6%)	345 (85.8%)	188 (39.3%)	
Surgical period				<0.001
2015–2018	347 (39.4%)	57 (14.2%)	290 (60.7%)	
2019–2022	188 (21.4%)	0 (0.0%)	188 (39.3%)	
2023–	345 (39.2%)	345 (85.8%)	0 (0.0%)	
Follow-up yrs, mean (SD)	1.0 (0.8)	0.9 (0.7)	1.1 (0.9)	0.008

Values are shown in mean (SD), otherwise indicated. Continuous variables were compared using a linear mixed model and categorical variables were compared using the chi-square test.

*BCVA* best-corrected visual acuity, *IOP* intraocular pressure, *MD* mean deviation, *PEA + IOL* phacoemulsification with intraocular lens implantation, *POAG* primary open-angle, *TMH* Tanito microhook, *VF* visual field, *XFG* exfoliation glaucoma.

Postoperative complications and safety outcomes are shown in Table 2. Hyphaema or retained blood occurred in 143 of 880 eyes (16.2%) and was more frequent in the ≥6 clock-hour group than in the <6 clock-hour group (102/478 [21.3%] vs 41/402 [10.2%]; *P* < 0.001). Hyphaema requiring treatment was also more frequent in the ≥6 clock-hour group (23/478 [4.8%] vs 2/402 [0.5%]; *P* < 0.001). IOP spike, fibrin reaction, and macular oedema were more frequent in the ≥6 clock-hour group. Additional glaucoma surgery occurred in 34 of 880 eyes (3.9%), with no statistically significant difference between incision groups (22/478 [4.6%] vs 12/402 [3.0%]; *P* = 0.215). No hypotony or loss of light perception was observed. BCVA loss of ≥2 lines at 12 months occurred in 15 of 441 eyes (3.4%) and did not differ significantly between incision groups (10/230 [4.3%] vs 5/211 [2.4%]; *P* = 0.252).

**Table 2:** Postoperative complications and safety outcomes stratified by incision extent.

Outcome	Overall	<6 clock hours	≥6 clock hours	*P* value
Hyphaema	143/880 (16.2)	41/402 (10.2)	102/478 (21.3)	<0.001
Hyphaema requiring treatment	25/880 (2.8)	2/402 (0.5)	23/478 (4.8)	<0.001
IOP spike	36/880 (4.1)	10/402 (2.5)	26/478 (5.4)	0.028
Hypotony	0/880 (0.0)	0/402 (0.0)	0/478 (0.0)	–
Fibrin reaction	29/880 (3.3)	6/402 (1.5)	23/478 (4.8)	0.006
Macular oedema	16/880 (1.8)	1/402 (0.2)	15/478 (3.1)	0.001
Ciliary body detachment	2/880 (0.2)	0/402 (0.0)	2/478 (0.4)	0.194
Vitreous haemorrhage	2/880 (0.2)	0/402 (0.0)	2/478 (0.4)	0.194
Additional glaucoma surgery	34/880 (3.9)	12/402 (3.0)	22/478 (4.6)	0.215
Loss of light perception	0/880 (0.0)	0/402 (0.0)	0/478 (0.0)	-
BCVA loss ≥2 lines at 12 months	15/441 (3.4)	5/211 (2.4)	10/230 (4.3)	0.252

Values are presented as n/N (%). *P* values compare the two incision extent groups and were calculated using chi-square tests. Hyphaema was defined using recorded postoperative hyphaema or blood retention fields. Hyphaema requiring treatment included anterior chamber washout, tissue plasminogen activator use, or recorded hyphaema treatment.

**Fig. 1:**
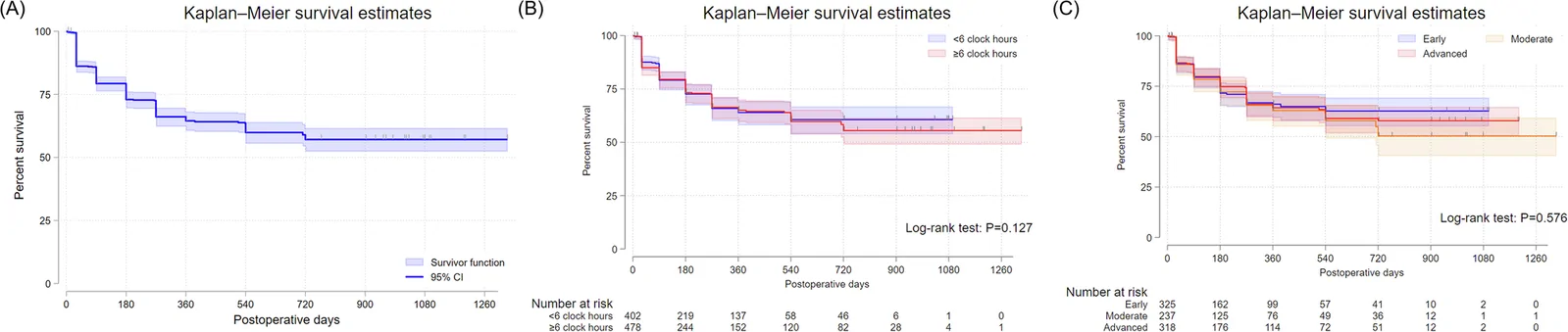
Kaplan–Meier survival curve. **A** Kaplan-Meier survival curve for surgical success in the overall cohort. By AAO MIGS criteria, cumulative success was 64.5% (95% CI, 60.6–68.2) at 12 months and 59.0% (95% CI, 54.4–63.2) at 24 months. Shaded bands indicate 95% confidence intervals. **B** Kaplan-Meier survival curves for surgical success stratified by incision extent ( < 6 clock hours and ≥6 clock hours). Shaded bands indicate 95% confidence intervals. There was no significant difference in cumulative success between incision-extent groups (log-rank *P* = 0.127). **C** Kaplan-Meier survival curves for surgical success stratified by baseline glaucoma severity (early, moderate, and advanced). Shaded bands indicate 95% confidence intervals. There was no significant difference in cumulative success across severity groups (log-rank *P* = 0.576).

By AAO MIGS criteria, cumulative success was 64.5% (95% CI, 60.6–68.2) at 12 months and 59.0% (95% CI, 54.4–63.2) at 24 months (Fig. 1A). Kaplan-Meier curves for the <6 and ≥6 clock-hour groups overlapped, with no significant difference by log-rank test (*P* = 0.127) (Fig. 1B). In the calendar-year-adjusted multivariable Cox model, ≥6 clock hours was not significantly associated with surgical failure compared with <6 clock hours (adjusted HR, 1.14; 95% CI, 0.85 to 1.53; *P* = 0.367) (Table 3). Similar findings were observed using the alternative IOP-defined failure criteria A–D (Supplementary Fig 1).

**Table 3:** Multivariable cox regression analysis for surgical failure.

Variable	Adjusted hazard ratio (95% CI)	*P* value
Incision extent <6 clock hours ≥6 clock hours	reference 1.14 (0.85–1.53)	– 0.367
Age, per year	0.99 (0.98–1.01)	0.465
Female sex	0.97 (0.75–1.24)	0.792
XFG vs POAG	0.97 (0.66–1.42)	0.863
Baseline IOP, per mmHg	0.93 (0.89–0.98)	0.002
Baseline VF MD, per dB	0.98 (0.97–1.00)	0.046
Baseline medications, per medication class	0.84 (0.76–0.93)	0.001
Surgical year	1.01 (0.97–1.06)	0.553

Hazard ratios were estimated using a multivariable Cox proportional hazards model for the primary surgical failure endpoint. The model included incision extent, age, sex, glaucoma diagnosis, baseline intraocular pressure, baseline 24-2 visual field mean deviation, baseline number of glaucoma medications, and surgical year. Robust standard errors were clustered by patient.

*HR* hazard ratio, *CI* confidence interval, *IOP* intraocular pressure, *VF MD* visual field mean deviation.

Baseline glaucoma severity was not significantly associated with surgical success by Kaplan-Meier analysis using the AAO MIGS criteria (log-rank *P* = 0.576) (Fig. 1C). Under the alternative IOP-defined criteria, survival differed across severity groups for definitions A and B but not for definitions C and D (Supplementary Fig 2). In the multivariable Cox model, lower baseline IOP was associated with a higher risk of surgical failure. Each 1-mmHg higher baseline IOP was associated with a lower hazard of failure (adjusted HR, 0.93; 95% CI, 0.89–0.98; *P* = 0.002) (Table 3).

**Fig. 2:**
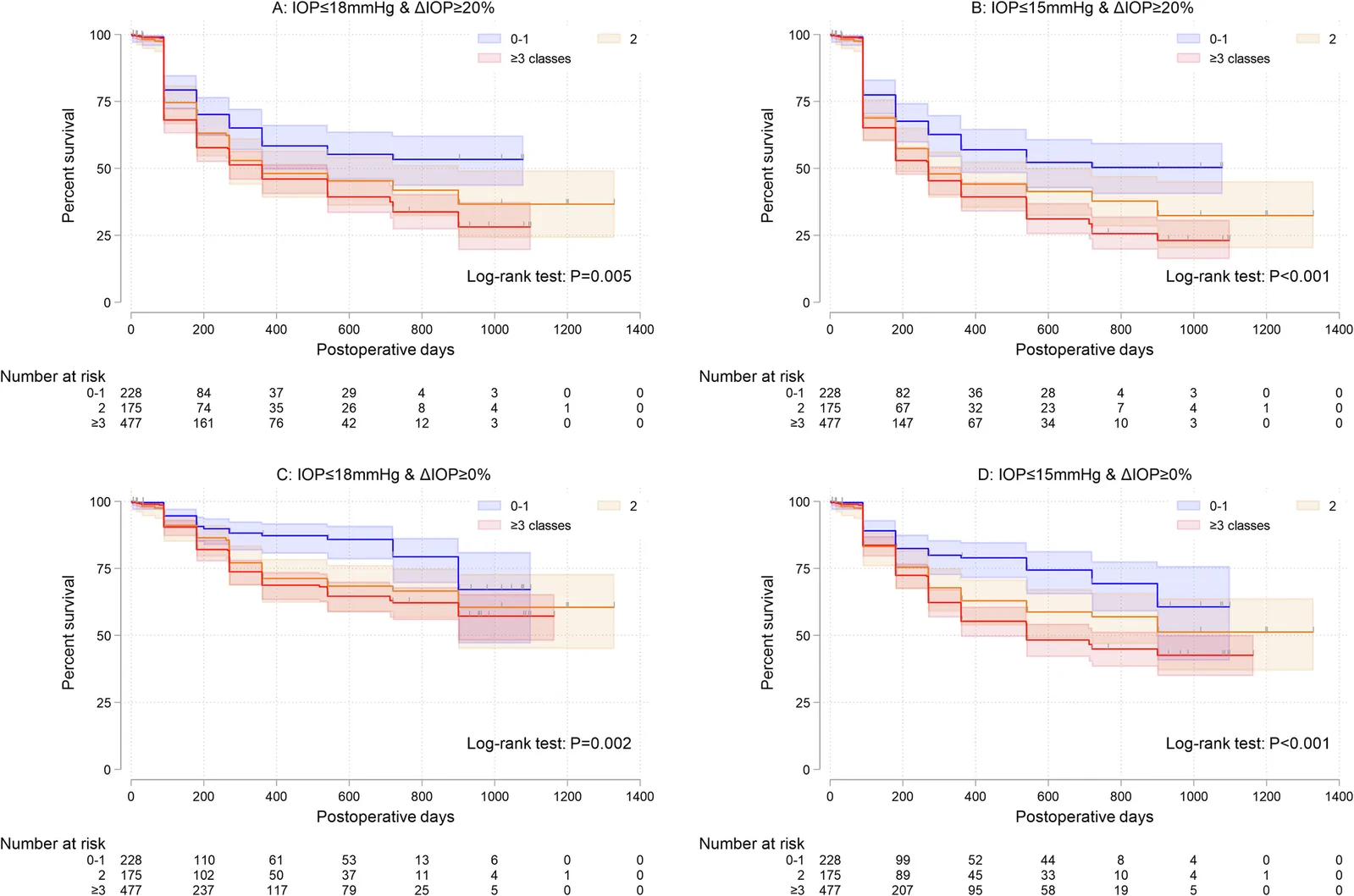
Kaplan-Meier survival curves stratified by baseline number of medication classes under four IOP-defined qualified success criteria. Criterion A required IOP ≤ 18 mmHg and ≥20% IOP reduction from baseline; criterion B required IOP ≤ 15 mmHg and ≥20% IOP reduction; criterion C required IOP ≤ 18 mmHg and no postoperative IOP increase above baseline; and criterion D required IOP ≤ 15 mmHg and no postoperative IOP increase above baseline. Failure was defined as not meeting the corresponding criterion, undergoing additional glaucoma surgery, or loss of light perception. Antiglaucoma medication use was allowed in all four criteria. Shaded bands indicate 95% confidence intervals. Survival differed across medication-class categories in all panels, with log-rank *P* = 0.005 for (**A**), *P* < 0.001 for (**B**), *P* = 0.002 for (**C**), and *P* < 0.001 for (**D**).

Across the four IOP-defined success definitions, higher baseline number of medications was associated with worse surgical success. Survival differed significantly across baseline medication classes (0–1, 2, and ≥3), with log-rank *P* = 0.005 for definition A, P < 0.001 for definition B, *P* = 0.002 for definition C, and *P* < 0.001 for definition D (Fig. 2). When stratified by baseline number of medications, postoperative medication trajectories differed across strata. In the 0–1 drop group, mean (SD) medication count increased modestly from 0.8 (0.4) at baseline to 1.1 (1.1) at 12 months and 1.3 (1.2) at 24 months. In the 2-drop group, mean medication count decreased to 1.8 (1.0) at 12 months and 1.4 (0.9) at 24 months. In the ≥3-drop group, medication use decreased after surgery but remained higher than in the other categories, from 3.5 (0.7) at baseline to 2.7 (1.1) at 12 months and 2.8 (1.0) at 24 months.

## Discussion

In this study, phacoemulsification combined with Tanito microhook trabeculotomy was associated with sustained reductions in both IOP and medication burden through 24 months, and the two-year cumulative success by the AAO MIGS criteria was 59.0%. In stratified and multivariable analyses, surgical failure did not differ between the <6 and ≥6 clock-hour incision groups after adjustment for surgical year and baseline covariates. Baseline glaucoma severity did not stratify survival, while higher baseline medication burden was associated with worse survival across IOP-defined criteria.

The overall direction and durability of IOP and medication reduction are consistent with prior studies of microhook-based ab interno trabeculotomy performed with cataract surgery and with comparative studies of trabecular MIGS with cataract surgery [14, 15]. However, direct comparisons of cumulative success across studies should account for key differences in trial design and baseline IOP ascertainment. Several pivotal randomised trials required medicated IOP to be controlled within a prespecified upper limit at screening (e.g., ≤24 mmHg), while also requiring substantially higher washed-out diurnal IOP at baseline, typically in the low-to-mid 20 s or higher, and assessed primary endpoints using unmedicated diurnal IOP after medication washout at follow-up [16–18]. In contrast, baseline IOP in the present cohort was measured under routine care without protocolised washout and was lower on treatment, and postoperative medication adjustments were made clinically rather than by trial protocol. These differences can make achieving a 20% or greater IOP reduction and maintaining success under IOP-based or medication-sensitive definitions more challenging, even when absolute IOP reductions are clinically meaningful.

A central issue in interpreting MIGS outcomes is the definition of surgical success. Success estimates can vary substantially depending on whether endpoints use IOP thresholds, relative IOP reduction, medication reduction, or complete versus qualified success [19]. This heterogeneity can produce markedly different reported success rates even within the same cohort. Recent guidance from the AAO was published specifically to standardise reporting, and for combined cataract-MIGS procedures it explicitly incorporates medication reduction as one pathway to success [5]. Accordingly, the present study used the AAO MIGS endpoint as the primary outcome and reported IOP-defined criteria A–D as sensitivity analyses to facilitate comparison with prior trabeculotomy and angle-surgery studies. These alternative IOP-defined analyses should be interpreted as exploratory rather than confirmatory.

Across the incision-extent categories, survival curves overlapped between the <6 and ≥6 clock-hour groups. In the calendar-year-adjusted multivariable Cox model, ≥6 clock hours was not significantly associated with surgical failure compared with <6 clock hours. This finding should not be interpreted as evidence that incision extent has no biological relevance. Rather, incision extent was correlated with calendar time and surgeon practice patterns. In addition, the physiologic benefit of extending the treated arc may be constrained by resistance distal to Schlemm’s canal and by episcleral venous pressure, potentially attenuating incremental gains once a sufficient portion of the trabecular pathway is opened [20, 21]. Consistent with this concept, prior study has shown that extent of trabeculotomy was not associated with postoperative IOP levels [13]. Another study, using propensity score–matched comparison of phaco-TMH with narrower (120°) versus wider (240°) incisions found similar IOP and medication outcomes, while early postoperative hyphaema scores were higher with the wider incision [22]. A similar pattern has been described with gonioscopy-assisted transluminal trabeculotomy (GATT), where segmental (180°) and circumferential (360°) procedures achieved comparable IOP and medication reductions and surgical success, but 360° GATT was associated with higher rates of hyphaema and IOP spikes [23, 24]. In the present cohort, hyphaema, hyphaema requiring treatment, IOP spike, fibrin reaction, and macular oedema were more frequent in the ≥6 clock-hour group. These findings suggest that extending the treated arc may increase early postoperative inflammatory or bleeding-related events, even if it does not translate into a clear reduction in surgical failure.

Baseline glaucoma severity did not stratify survival in this study. Although angle-based MIGS performed with cataract surgery is most commonly positioned for mild-to-moderate glaucoma [25], observational studies and real-world cohorts have reported outcomes in advanced, severe, or refractory disease across several trabecular procedures, including Kahook Dual Blade, GATT, and Trabectome [26–29]. These studies suggest that worse glaucoma severity does not necessarily translate into a proportional loss of sustained surgical success after trabecular MIGS. Finally, the broader MIGS evidence base remains shaped by pivotal trials that enrol mild-to-moderate disease, which improves interpretability and safety but limits direct generalisability to advanced glaucoma.

Baseline IOP was also relevant to the interpretation of surgical failure. In the multivariable model, lower baseline IOP was associated with a higher risk of meeting the failure endpoint. This finding is expected because eyes starting with lower IOP have less room to achieve percentage-based IOP reduction, even when postoperative IOP is clinically acceptable. Therefore, comparisons of success rates across studies or subgroups should account for baseline IOP and whether endpoints require relative IOP reduction.

In contrast, baseline number of glaucoma medications was the clearest discriminator of outcome across the IOP-defined success definitions. A higher preoperative medication burden may reflect more limited outflow reserve or a lower target IOP requirement, which could reduce the likelihood of meeting IOP-defined success after trabecular surgery [20, 21] This observation is also relevant to endpoint selection. The AAO-recommended success definition for MIGS combined with cataract surgery allows success to be met either through IOP-based criteria or through a reduction of at least one medication without an IOP increase, so estimated success can vary with baseline medication burden and postoperative medication management. Accordingly, subgroup survival comparisons were based on the IOP-defined success definitions, and medication use was reported separately to support comparability across strata defined by baseline number of medications. The modest postoperative increase in medication use in the 0–1 drop group likely reflects real-world treatment escalation in eyes that did not reach the desired postoperative target IOP, rather than a direct adverse effect of trabeculotomy. Because postoperative medication changes were not protocolised, these trajectories should be interpreted as clinical management patterns.

This study has limitations. First, the retrospective design introduces the potential for unmeasured confounding, and incision extent was correlated with calendar time and surgeon practice patterns. Although the multivariable Cox model adjusted for surgical year and baseline covariates, residual confounding by surgeon-specific technique, postoperative management, or secular changes in care remains possible. Second, postoperative medication escalation and decisions regarding additional procedures were made in routine clinical care rather than by protocol, which may influence survival endpoints, particularly when medication use is incorporated into the failure definition. Third, incision extent was based on intraoperative estimation and was not independently verified by standardised postoperative gonioscopy. Therefore, the clock-hour categories should be interpreted as estimated treated extent rather than exact anatomic measurements. Fourth, follow-up availability decreased over time, and 24-month outcomes were available for a smaller subset of eyes. Fifth, alternative failure definitions and subgroup analyses were evaluated without multiplicity adjustment; therefore, borderline *P* values should be interpreted cautiously and should not be considered confirmatory. Strengths include the large sample size, scheduled postoperative assessment time points, and the use of both AAO MIGS criteria and commonly used IOP-based alternative definitions to improve comparability with prior MIGS studies.

In conclusion, phacoemulsification combined with TMH achieved sustained reductions in IOP and medication burden through 24 months. Surgical failure did not differ by incision extent after adjustment for surgical year and baseline covariates. Lower baseline IOP was associated with a higher likelihood of meeting the primary failure endpoint, whereas higher baseline medication burden was associated with poorer IOP-defined success. These findings support considering baseline IOP, medication burden, and endpoint definition when counselling patients and comparing trabecular MIGS outcomes.

## Summary

### What was known before

- Phacoemulsification combined with minimally invasive glaucoma surgery (MIGS), including trabeculotomy-based procedures, can reduce intraocular pressure (IOP) and glaucoma medication burden, but reported success rates vary depending on the success definition and baseline disease characteristics.
- It remains unclear whether preoperative and intraoperative factors, including incision extent, baseline glaucoma severity, and baseline medication burden, meaningfully differentiate surgical success for MIGS combined with cataract surgery.

### What this study adds

- In a large cohort of 880 open-angle glaucoma eyes undergoing Tanito microhook trabeculotomy combined with cataract surgery (phaco-TMH), IOP and medication burden improved through 24 months.
- Surgical success did not differ by incision extent or baseline glaucoma severity, while higher baseline medication burden was associated with worse IOP-defined success across multiple alternative success definitions, suggesting that baseline medication burden can be a preoperative marker of a lower probability of sustained IOP-defined success after phaco-TMH.

## Supplementary information

Supplemental Figure 1

Supplemental Figure 2

Supplementary Table 1

## Data Availability

The data that support the findings of this study are available from the corresponding author upon reasonable request.
