# Supplementary material for: Outcomes of phacoemulsification combined with ab interno trabeculotomy: stratified by incision extent, glaucoma severity, and baseline number of medications
Source: Eye (Lond). 2026 Jul 22;40(15):2417–23. doi: 10.1038/s41433-026-04749-x (PMC13675221; doi:10.1038/s41433-026-04749-x)
Supplement: Supplementary file 1 [file 41433_2026_4749_MOESM1_ESM.docx]

**Supplemental Figure 1**

Kaplan–Meier survival curves for surgical success stratified by incision extent (<6 clock hours and ≥6 clock hours) under four IOP-defined success definitions (A–D). Survival did not differ significantly across incision-extent groups (log-rank P = 0.659 for A, P = 0.711 for B, P = 0.173 for C, and P = 0.087 for D, respectively).
