# Supplementary material for: Outcomes of phacoemulsification combined with ab interno trabeculotomy: stratified by incision extent, glaucoma severity, and baseline number of medications
Source: Eye (Lond). 2026 Jul 22;40(15):2417–23. doi: 10.1038/s41433-026-04749-x (PMC13675221; doi:10.1038/s41433-026-04749-x)
Supplement: Supplementary file 2 [file 41433_2026_4749_MOESM2_ESM.docx]

**Supplemental Figure 2**

Kaplan–Meier survival curves for surgical success stratified by baseline glaucoma severity (early, moderate, and advanced) under four IOP-defined success definitions (A–D). Survival differed across severity groups for definitions A and B, but not for definitions C and D (log-rank P = 0.004 for A, P = 0.035 for B, P = 0.502 for C, and P = 0.977 for D, respectively).
