# Supplementary material for: Outcomes of phacoemulsification combined with ab interno trabeculotomy: stratified by incision extent, glaucoma severity, and baseline number of medications
Source: Eye (Lond). 2026 Jul 22;40(15):2417–23. doi: 10.1038/s41433-026-04749-x (PMC13675221; doi:10.1038/s41433-026-04749-x)
Supplement: Supplementary file 3 [file 41433_2026_4749_MOESM3_ESM.docx]

| **Supplementary Table 1. Definitions of Surgical Failure Criteria** | | | | | |
| --- | --- | --- | --- | --- | --- |
| **Definition** | **Timing** | **Medication condition** | **IOP threshold criterion** | **IOP reduction / baseline criterion** | **Other failure criteria** |
| AAO endpoint | ≥30 days | Medication reduction or no medication increase, depending on success component | IOP ≤21 mmHg for the IOP-reduction success component | Success if ≥1 medication reduction without IOP increase, or IOP ≤21 mmHg with ≥20% IOP reduction and no medication increase | Hypotony, additional glaucoma surgery, or loss of light perception counted as failure |
| Criterion A | >90 days postoperatively | Antiglaucoma medication use allowed | IOP >18 mmHg | <20% IOP reduction | Additional glaucoma surgery or loss of light perception |
| Criterion B | >90 days postoperatively | Antiglaucoma medication use allowed | IOP >15 mmHg | <20% IOP reduction | Additional glaucoma surgery or loss of light perception |
| Criterion C | >90 days postoperatively | Antiglaucoma medication use allowed | IOP >18 mmHg | IOP above baseline | Additional glaucoma surgery or loss of light perception |
| Criterion D | >90 days postoperatively | Antiglaucoma medication use allowed | IOP >15 mmHg | IOP above baseline | Additional glaucoma surgery or loss of light perception |
| Definitions A–D followed the IOP-based qualified success criteria reported by Tanito et al. Failure was assessed after 90 postoperative days and included IOP exceeding the specified threshold, insufficient IOP reduction from baseline, IOP above baseline where applicable, additional glaucoma surgery, or loss of light perception. Antiglaucoma medication use was allowed. | | | | | |
